# The HTLV-1 HBZ Oncoprotein and Its Role in Adult T-Cell Leukemia/Lymphoma

**DOI:** 10.3390/cancers18132101

**Published:** 2026-06-28

**Authors:** Roberto S. Accolla, Mariam Shallak, Greta Forlani

**Affiliations:** Laboratories of General Pathology and Immunology “Giovanna Tosi”, Department of Medicine and Technological Innovation, University of Insubria, 21100 Varese, Italy; mariam.shallak@uninsubria.it

**Keywords:** HTLV-1, human retroviruses, ATL, HBZ, Tax-1, leukemia

## Abstract

Human T-cell leukemia virus-1 (HTLV-1) is the etiological agent of a series of chronic inflammatory diseases such as HTLV-associated myelopathy/Tropical spastic paraparesis (HAM/TSP), uveitis, dermatitis, and pneumonitis, and, importantly, of a T-cell lymphoproliferative neoplasm designed adult T-cell leukemia/lymphoma (ATL). Two viral proteins, Tax-1 and HBZ, are crucially involved in HTLV-1 infectivity and in ATL by altering key pathways of cell homeostasis. A fundamental distinction between the expression of the two oncoproteins exists, witnessed by the fact that Tax-1 is expressed in early phases of HTLV-1 infectivity and ATL onset but may be lost in a substantial number of established ATL, whereas HBZ is always expressed in all phases of HTLV-1 infection and in all ATL. Additionally, while Tax-1 can be localized both in the cytoplasm and nucleus in all cases of disease, recent evidence indicate that HBZ is localized solely in the cytoplasm in cells of HTLV-1-infected individuals, asymptomatic carriers (AC) and patients suffering from HAM/TSP. Importantly, ATL instead marks a progressive dislocation of HBZ in the nucleus. Thus, both the expression and the subcellular localization of HBZ represent distinctive elements in the process of HTLV-1-associated pathology. Within this frame, recent studies point to a very important involvement of HBZ in disarranging the homeostasis of the cell not only at the transcriptional but most importantly at the post-transcriptional level as a result of the interaction with crucial factors regulating RNA splicing and stability. These recent aspects of the HBZ biology will be discussed for their implication in HTLV-1-mediated oncogenesis.

## Simple Summary

HTLV-1 is a human oncogenic virus that is responsible for a severe form of adult T-cell leukemia-lymphoma (ATL). Two viral products are strongly involved in the development of ATL, the viral transactivator Tax-1 and the HTLV-1 basic leucine zipper (HBZ) protein. HBZ is the focus of this review. We will describe the discovery, the cell biology, and the recent findings that point to the complex mechanism through which HBZ may exert its oncogenic activity. Through these studies, novel unprecedented information seems to emerge on the action of HBZ on basic post-transcriptional mechanisms related to mRNA stability and splicing.

## 1. Introduction

HTLV-1 (Human T cell Leukemia/Lymphoma type 1 virus) is the first human oncogenic retrovirus discovered [1,2]. Infection by HTLV-1 is endemic in distinct areas of the world that include the southern part of Japan, the Caribbean, North and South America, Central and West Africa, restricted parts of Middle East, Australia, and Melanesia [3]. The virus induces clonal proliferation of infected cells and this enhances its propagation, allowing the virus to be transmitted primarily by cell-to-cell contact [4,5,6].

HTLV-1 is the etiological agent of a series of inflammatory diseases among which HTLV-1-associated myelopathy/tropical spastic paraparesis (HAM/TSP) represents one of the most severe and debilitating pathological states [7,8]. As an oncogenic virus, HTLV-1 is the causative agent of a very serious form of blood cancer designated adult T cell leukemia/lymphoma or ATL, whose development may affect 3–5% of infected patients and may require several decades from primary infection to develop [9]. The reason for the very late development of ATL is not clear but it may correlate in part to the long period of viral latency that often occurs after infection to the clinical manifestation of the disease. Recently, the existence of an intragenic viral silencer in an open chromatin region within the HTLV-1 provirus has been reported. This sequence acts as a binding region of the host transcription factor RUNX1, which then represses viral replication. In this way, this viral silencer can regulate viral latency, explaining in a way why HTLV-1 after integration persists without giving rise to active replication. This viral silencer transferred to the HIV genome induces HIV-1 viral latency as well [10].

The extremely high incidence of ATL compared to other cancers with a virus etiology makes HTLV-1 the most oncogenic pathogen to date [11]. Sadly, at present, ATL still lacks a definitive cure and patients often succumb after a short period from diagnosis [12]. The HTLV-1-dependent disarrangement of the cell homeostasis leading to neoplastic transformation has been mainly attributed to two viral products, the viral transactivator Tax-1 and the HTLV-1 basic leucine zipper (HBZ) proteins. Tax-1 is expressed early during infection and has been shown to alter basic mechanisms of the cell such as proliferation, DNA damage repair, and apoptosis [13,14,15,16]. The oncogenic properties of Tax-1 are strongly associated with its ability to constitutively activate the nuclear factor kappa B (NF-kB) pathway [17,18,19]. Importantly, however, genetic and epigenetic modifications in the proviral genome often occur leading to Tax-1 silencing [20]. Indeed 60% of ATL patients do not express Tax-1 mRNA suggesting that this viral factor is important in the onset of neoplastic transformation but is dispensable for the maintenance of leukemia [14]. More recent evidence, however, suggests a more complex mechanism for the involvement of Tax-1 in ATL, at least in those cases in which the Tax-1 coding gene is not deleted or structurally modified. Indeed, it has been shown that Tax-1 is expressed intermittently in a small proportion of ATL cells in a cell cycle-dependent fashion [21] and this sporadic on/off switching in expression may be crucial in maintaining the whole population of virus-induced leukemic cells [22]. This is also confirmed by the recent observation that ATL cells may still make small amounts of Tax mRNA and proteins and appear to still be dependent on Tax-1 [23].

Tax-1 is a highly immunogenic protein and this makes it a major target of cytolytic T cell lymphocytes (CTL). This fact has been interpreted as a major force leading to the loss of Tax-1 expression during the protracted clinically latent period leading to overt ATL [24].

## 2. HTLV-1 HBZ

**Figure 1:**
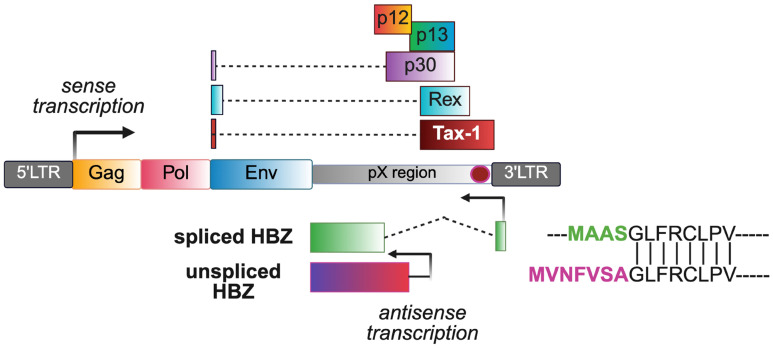
Schematic representation of HTLV-1 proviral genome organization and transcriptional activity. Sense transcription initiated at the 5′ long terminal repeat (5′LTR) produces canonical viral transcripts encoding structural and regulatory proteins, including Gag, Pol, Env, and the pX region products (Tax-1, Rex, p30, p13, and p12), as indicated by dashed lines. Antisense transcription originates from the 3′LTR and gives rise to the HBZ transcripts, which are expressed as both spliced and unspliced isoforms, generating proteins with distinct N-terminal sequences. The red circle marks a viral enhancer element located in the 3′LTR region that is required for efficient antisense transcription of the integrated provirus.

Unlike Tax-1, HBZ is always expressed during HTLV-1 infection and stays stably expressed in ATL cells. HBZ counteracts Tax-activated viral and cellular pathways and stimulates cell proliferation [25]. Originally described by Larocca et al., [26], HBZ is transcribed from the anti-sense strand of the viral genome [27] (Figure 1). The latter characteristic is of primary importance because for many years the existence of anti-sense transcripts and related proteins made by retroviruses has been overlooked, if not ignored. Thus, the paradigm of retroviruses encoded proteins by only one strand of proviral DNA has shifted, mostly because of the discovery of HBZ [28]. In an attempt to explain the constitutive expression of HBZ, Matsuo and coll. have attentively studied viral sequences that may contribute to this constitutive expression and found a viral enhancer, that is a specific viral sequence conferring increased downstream transcriptional activity, that indeed acts as a regulatory hotspot driving continuous and persistent transcription of the anti-sense provirus [29].

As far as the structure, HBZ contains a bZIP domain at the C-terminus, an activation domain at the N-terminus and a central domain [27]. HBZ is expressed under three distinct isoforms: two alternative spliced forms (SP1 and SP2) and an unspliced (us) form (Figure 1) [30,31]. SP1 occurs more frequently than SP2. SP1 differs from the SP2 isoform for a shorter and distinct sequence at exon 1, generating a protein of 206 aa versus a potential 216 aa protein for SP2. However, only SP1 has been detected at the protein level. Thus, the real function of SP2 is still elusive. The sequences of SP1 and usHBZ forms are identical with the exception of the first 7 amino acids, giving rise to a protein of 209 amino acids for usHBZ. Although the two protein variants exhibit similar functions [32], the spliced form is more abundant than the unspliced form and is found in almost all ATL patients [33].

While the involvement of Tax in triggering oncogenesis has been widely known for long time [19,34] a similar action of HBZ was supposed later on the basis of several evidences including the elegant observation of Satou and coll. who showed that HBZ induced a T cell lymphoma in vivo in transgenic mice [35].

## 3. HBZ Subcellular Localization

As part of HBZ counteracting Tax-activated viral and cellular pathways, it should be underlined the crucial function of HBZ in suppressing Tax-1-induced transcription from the 5′LTR in part via the block of LTR recruitment of several transcription factors used by Tax-1 such as ATF/CREB and CBP/p300 to activate viral genome transcription [25]. As these factors are localized in the nucleus, it was assumed for long time that HBZ was an exclusive nuclear protein [36].

The isolation of the first described monoclonal antibody against HBZ by our group allowed us to precisely quantitate, localize in subcellular compartments, and assess the interaction in vivo with several previously reported transcription factors involved in the metabolic modification of HTLV-1-infected cells [37]. It was found that the amount of HBZ expressed in fresh leukemic cells was in the order of 20-to-50-fold less than the amount expressed in HBZ-transfected cells, giving a more real picture of the “physiological” expression of the viral protein in the real world. Moreover, those first studies helped to better clarify some aspects of the in vivo interaction between HBZ and nuclear factors such as p300, CBP, JunD, and CREB2, demonstrating a partial colocalization of HBZ with them, depending on the amount of expressed viral protein both in chronically infected and ATL leukemic cells [37]. The availability of a reagent that could detect HBZ in vivo also offered a potent tool to finally assess the subcellular distribution of HBZ in the various conditions from primary infection to disease states including inflammatory HAM/TSP and ATL. In contrast to previous belief, HBZ was found to localize exclusively in the cytoplasm of infected cells of asymptomatic carriers (AC), which could express the protein in 1–4% of peripheral blood mononuclear cells (PBMC). Similarly, and importantly, HBZ was also strictly localized in the cytoplasm in the PBMC of HAM/TSP patients, in this case in a greater percentage of cells (up to 10%). This defined for the first time the existence and the location of a marker of infection associated with progression to the neuroinflammatory disease [38,39,40]. Importantly, the cytoplasmic expression of HBZ was a stable feature of the protein since it was additionally shown that it could not shuttle between cytoplasm and nucleus as assessed by treatment with Leptomycin B (LMB), a specific inhibitor of CRM1/exportin-mediated nuclear export [41].

Of great interest, in both HAM/TSP and AC, the expression of HBZ and Tax-1 was rarely found in the same cell, suggesting the existence of mechanisms of expression uncoupling of these viral oncogenes. Whether this observed uncoupling has implications for the evolution of HTLV-1-associated infection toward overt disease is still a matter of debate [42]. CD4+ T cells are the major subcellular compartment expressing cytoplasmic HBZ, with very few CD8+ T cells positive for the marker and other cell subpopulations, including B cells and NK cells totally negative (40).

It has been shown that HTLV-1 can infect CD4+ T cells with a CD25+ suppressor phenotype, the so called regulatory T cells or Tregs [43], in which an impaired function was observed, particularly evident in HAM/TSP patients that have a high number of Tregs and higher HBZ mRNA levels [44,45]. It was then possible that the cytoplasmic HBZ protein detected in AC and HAM/TSP patients could be preferentially found in CD4+/CD25+ Tregs. A more detailed analysis of the CD4+ cell phenotype showed that this was not always the case, thus suggesting the possible uncoupling of HBZ mRNA and protein expression in Tregs as well as in other CD4+ T cell subpopulations in both AC and HAM/TSP patients [43,45,46,47].

**Figure 2:**
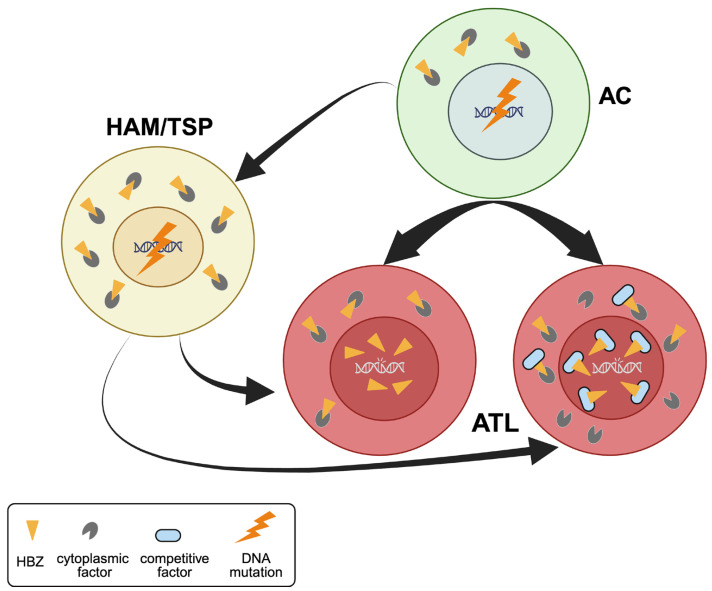
Model of HTLV-1–associated tumor progression and HBZ subcellular relocalization. Schematic representation of the proposed model linking HTLV-1 infection outcomes to HBZ localization. In asymptomatic carriers (AC) and in HAM/TSP, HBZ is predominantly retained in the cytoplasm through interaction with a cytoplasmic anchoring factor. During progression toward adult T-cell leukemia (ATL), HBZ undergoes relocalization from the cytoplasm to the nucleus, where it exerts its transcriptional functions. This transition is hypothesized to be driven by mutation-associated events at the proviral DNA level or by epigenetic changes, known to be important in ATL, that promote nuclear accumulation of HBZ through two non-mutually exclusive mechanisms: (i) reduction or loss of the cytoplasmic retention factor, resulting in decreased cytoplasmic sequestration of HBZ; or (ii) induction of a competing factor that binds HBZ and facilitates its nuclear translocation. The nuclear enrichment of HBZ is proposed to contribute to leukemogenic progression.

The analysis of HBZ subcellular localization in fresh cells of leukemic patients showed, instead, the crucial difference with AC and HAM/TSP in that HBZ was present both in the cytoplasm and the nucleus irrespective of the stage of the disease, as the same pattern was observed in the cells of acute and chronic ATL patients [48]. Thus, for the first time it could be demonstrated that the oncogenic transformation due to HTLV-1 infection was marked by the transition of HBZ protein from cytoplasm to the nucleus [48]. Interestingly, however, even in ATL cells the HBZ molecular pool was not freely shuttling between the cytoplasm and nucleus as previously supposed [49], strongly suggesting that a cytoplasmic factor could retain HBZ in the cytoplasm all through the life history of HTLV-1 infection until neoplastic transformation occurs from which a progressive release of HBZ and its transition to the nucleus takes place. This could happen either because the cytoplasmic factor is reduced or because additional competitive factors could bind HBZ and allow its migration and retention in the nucleus [48] (Figure 2). Following our studies, Zhang et al. have also analyzed the subcellular HBZ distribution and confirmed our data on the difference between cytoplasmic and nucleus distribution between HTLV-1-infected and ATL cells, respectively. They also found that after TGF-β activation, translocation of HBZ protein from the cytoplasm to the nucleus was observed in ATL patients, but not in HTLV-1 carriers [50], further reinforcing the concept of a neoplastic action of HBZ only after its migration to the nucleus. Recent studies have shown that once in the nucleus, HBZ preferentially localizes in the nucleolus where it is found to be associated with Nucleophosmin/B23, a nucleolar phosphoprotein with multiple functions. This nucleolar localization potentially allows HBZ to interact with additional nucleolar proteins with proliferative and immortalizing effect [51].

The above findings leave the question of the functional role of the exclusive cytoplasmic distribution of HBZ in non-cancer cells unresolved. Of course, cytoplasmic HBZ could not be inert, bound to inhibitors, waiting to be displaced into the nucleus in leukemic cells. One possible role could be the modulation of cellular signaling leading to increased proliferation and/or the modulation of the immune response against the virus favoring the latent state of infection. Future investigation into this very important issue certainly deserves special attention. Within this frame, assessing the cytoplasmic HBZ interactome will be a key point.

## 4. The HBZ Endogenous Interactome

In addition to the large body of studies suggesting that the HBZ nuclear localization exerts a pivotal role in the process of oncogenesis through direct and indirect actions [24], a complete and structured analysis aimed at assessing the whole landscape of endogenous HBZ interaction with host molecules, was still lacking. Recent work has focused on filling this gap. A first attempt to clarify in an unbiased way the HBZ interactome was performed by the Twizere group who tested binary interacting pairs between HBZ and human transcription factors and RNA binding proteins using an established binary interactome mapping strategy employing primary screening by yeast two-hybrid (Y2H) assays and validation using an orthogonal protein complementation assay [52]. Interestingly, they found correlation with 47 HBZ-interacting proteins involved in several aspects of RNA function including RNA processing and splicing. The above interesting studies suffered, however, from the limitation of assessing interactions in a cell free system, not taking into account of the real endogenous HBZ interactome of an HTLV-1-derived leukemic cell.

**Figure 3:**
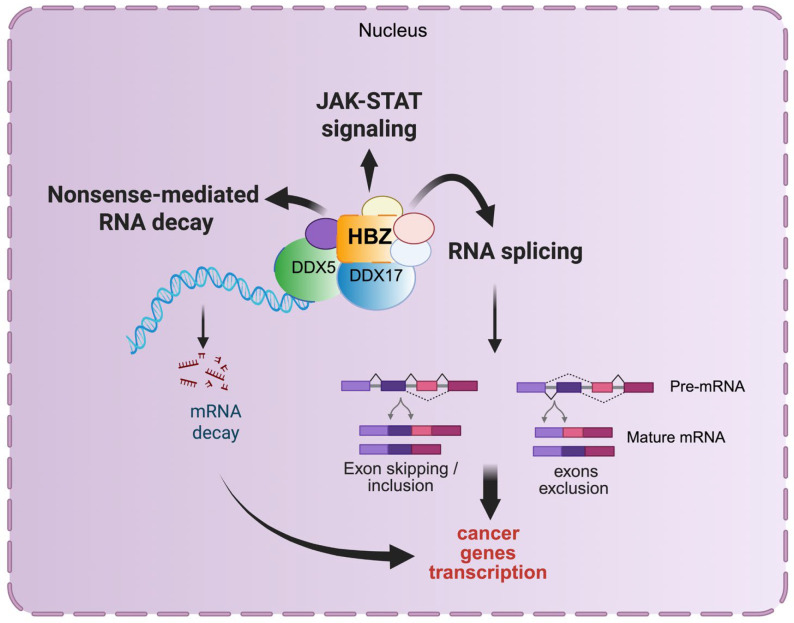
Proposed model of HBZ-mediated oncogenic transformation. Schematic representation of the molecular mechanisms through which HBZ may promote neoplastic transformation in HTLV-1–infected cells. The HBZ interactome is predominantly enriched in proteins involved in RNA splicing, RNA surveillance and decay pathways, particularly nonsense-mediated mRNA decay (NMD), and activation of the JAK-STAT signaling pathway. Among these interactors (round symbols), HBZ has been shown to associate with key RNA helicases and splicing regulators, including DDX5 and DDX17. Through these interactions, HBZ is hypothesized to perturb the stability and function of the RNA processing machinery, leading to widespread alterations in RNA splicing and mRNA turnover. This dysregulation may result in aberrant exon inclusion or exclusion (e.g., exon skipping), as well as altered RNA decay dynamics, thereby modulating the expression and isoform composition of critical oncogenes and tumor suppressor genes. In parallel, HBZ contributes to the activation of the JAK-STAT pathway, further promoting transcriptional programs associated with cell proliferation and survival. Collectively, these processes are proposed to drive oncogenic reprogramming and support leukemogenic progression.

Our group has investigated in detail the endogenous HBZ interactome by immunoprecipitating the HBZ-interacting complex in leukemic cells in conjunction with tandem mass spectrometric analysis [53]. It was found that the HBZ interactome was composed of at least 249 interactors encompassing three main clusters corresponding to molecular families involved in mRNA splicing, nonsense-mediated RNA decay, and JAK-STAT signaling pathway (Figure 3). While the implication of clusters related to JAK-STAT signaling and nonsense-mediated RNA decay will be matter of further studies, a detailed investigation of the cluster associated with RNA splicing indicated that interaction with HBZ altered the transcription of many genes including a large family of cancer-related genes, by affecting different splicing events such as exon skipping, both at the level of exon exclusion and exon inclusion. Importantly, RNA helicases DDX5 and DDX17, shown to be involved in alternative splicing of genes after NF-kB activation by Tax-1 [54], were two splicing factors that interacted and colocalized with HBZ (Figure 3). These studies are the first to depict the endogenous HBZ interactome and demonstrate an unprecedented role of the viral oncogene in the establishment of the oncogenic state [53].

## 5. HBZ as a Peculiar Example of a Bimodal Functional Gene

One of the most peculiar and rather unique features of HBZ is that it functions on two levels, both as a protein and as an mRNA molecule. The first evidence that HBZ mRNA could exert autonomous biological functions on disarranging cell homeostasis came from the work of Satou et al. who showed that a mutant gene allowing expression of HBZ mRNA but not translated protein was still able to support the proliferation of ATL cells [55]. Wild type gene coding for HBZ protein instead suppressed Tax-1-mediated viral transcription through the 5′ LTR. Their study also showed that the structure of HBZ mRNA, particularly the formation of secondary stem-loop structures, was crucial to maintaining proliferative capacity of ATL cells. In further studies it was also shown that the HBZ mRNA increased cell number by attenuating cell apoptosis [56]. Moreover, HBZ mRNA directly affects interaction of RNAPII with the LTR promoter by displacing TBP, the initiator of basal transcription. This mechanism impairs 5′LTR-directed gene expression of viral structural, enzymatic, and regulatory proteins. Because these viral proteins stimulate a vigorous cytotoxic and humoral immune response in HTLV-1-infected individuals, HBZ mRNA-dependent inhibition of sense transcription could be a key mechanism required for the persistence of infected cells thus favoring entry into latency and escape from immune response [57].

These features correlate with the strong tendency of HBZ mRNA to be retained in the nucleus and suggest a direct action of HBZ mRNA in the oncogenic process [58,59]. This idea is further supported by the finding that HBZ mRNA activates miRNAs with demonstrated oncogenic potential [60]. Despite the above findings, there is still not perfect concordance with the idea that HBZ mRNA is fully involved in the oncogenic process, as shown by recent comparative studies between HBZ protein and HBZ mRNA indicating the former playing a more critical role in establishing viral persistence and leukemogenesis in vivo in a humanized immune system mouse model [61].

## 6. Conclusions

HTLV-1-encoded HBZ represents an interesting and quite peculiar viral product that is involved in various aspects of HTLV-1 infectivity and virus-mediated diseases. Its constant expression during the life span of HTLV-1 infection, viral latency, disease progression, and cancerous state indicates the strong necessity by the virus to utilize the protein to maintain infectivity, to elude the immune response, and to be part of the process of neoplastic transformation. Its peculiar behavior in terms of subcellular localization constitutes from one side a sort of dynamic marker of disease association, being expressed solely in the cytoplasm of the cells of asymptomatic carriers and subjects with HAM/TSP neuroinflammation and in the cytoplasm and nucleus of the cells of leukemic patients, and from the other side a major actor in the maintenance of ATL, a devastating disease that still does not have a definitive cure. The discovery that HBZ may serve its functional activities both as protein and as mRNA adds a further element of unicity in the panorama of pathogen infections. All together, these features make future investigation on the biochemical and cellular aspects of endogenous HTLV-1 HBZ a very promising field for the advancement of knowledge of the biology of retrovirus infection, its associated diseases, and a better cure of them.

## Data Availability

Data supporting reported results can be found in the appropriate references included in the manuscript.

## Abbreviations

HTLV-1, Human T cell Leukemia-Lymphoma Virus-1; HBZ, HTLV-1 basic leucine zipper; Tax-1, HTLV-1 transactivator; ATL, Adult T cell leukemia-lymphoma; HAM/TSP, HTLV Associated Myelophaty/Tropical Spastic Paraparesis; AC, Asymptomatic Carriers; LTR, Long Terminal Repeat; PBMC, Peripheral Blood Mononuclear Cells; CTL, cytolytic T cell lymphocytes; LMB, Leptomycin B (LMB).
